# Impact of ultrasonic probe type, frequency, and static pressure on large-scale graphene exfoliation

**DOI:** 10.1016/j.ultsonch.2024.107103

**Published:** 2024-10-14

**Authors:** Minhui Gao, Hu Zong, Lei Yu, Jiacheng Yao, Su Zhao, Ying Zhou, Yifei Li, Yanyuan Zhou, Jiahao Zhang, Ronghe Li

**Affiliations:** a Ningbo Institute of Materials Technology and Engineering, Chinese Academy of Sciences, Ningbo 315201, China; b College of Chemical Engineering, Zhejiang University of Technology, Hangzhou 310014, China

**Keywords:** Ultrasound, Probe shape, Frequency, Pressure, Graphene

## Abstract

The ultrasonic liquid phase exfoliation method has emerged as an essential research direction for graphene preparation due to its cost-effectiveness and ability to minimize defects. However, this method faces challenges related to processing throughput when scaled up for industrial production. In this study, industrial grade ultrasonic homogenizers with different frequencies and probe types were evaluated for the preparation of FLG. In each experiment, 1.5 kg of graphite slurry was treated using a cyclic ultrasonic system. The results demonstrated that the 25 kHz dumbbell probe produced the thinnest FLG with the lowest defect density. Moreover, applying a static pressure of 0.2 MPa in the cycle system enhanced the cavitation-induced exfoliation of graphite sheets, effectively reducing the layer count and distribution range of FLG. This method improves the conductivity while minimizing defect density.

## Highlights

• The dumbbell probe enhances the preparation of thinner few-layer graphene (FLG).
• The 25 kHz probe yields the thinnest FLG (∼6 layers) with minimal defects.
• Applying 0.2 MPa pressure reduced the FLG layer count and narrowed its distribution.

## 1 Introduction

As one of the large-scale graphene preparation methods, ultrasonic liquid phase exfoliation overcomes the van der Waals force between graphene sheets by introducing ultrasonic waves [1]. Compared with other liquid phase exfoliation methods, such as high-pressure homogenization and supercritical CO_2_ liquid phase exfoliation, ultrasonic liquid phase exfoliating shows significant advantages for its low cost, easy operation, and potential scalability [2], [3]. The prepared graphene dispersions can be applied to conductive inks [4], anti-corrosion coatings [5], and heat dissipation materials [6].

In preparing graphene by ultrasonic liquid phase exfoliation, the shock waves and micro-jets generated by the collapse of cavitation bubbles effectively overcome the van der Waals forces between graphite layers, thereby obtaining FLG [7]. Consequently, various factors influencing the intensity of ultrasonic cavitation, including the liquid phase systems [8], [9], ultrasonic forms [10], frequency [11], power [12], and environmental pressure [13], are identified as critical in modulating the efficacy of the cavitation exfoliating process.

As a green and low-cost solvent [14], deionized water is for large-scale applications, especially when combined with effective dispersants, which helps to increase the concentration of graphene effectively [15]. Ultrasonic probes are more suitable for exfoliating graphite sheets than ultrasonic baths due to their high concentration of ultrasonic power and flexible design [16], [17]. Studies indicate that the dimensions and shapes of ultrasound probes impact the quality of exfoliated materials [10]. The resonant frequency of the ultrasonic system influences cavitation bubble size, affecting the exfoliating effect [18]. Previous research has shown that high-frequency ultrasound waves (∼1140 kHz) generate smaller bubbles and greater cavitation intensity, producing thinner and higher-quality graphene [14], [19].

However, it was shown that thin FLG can be prepared using low-frequency ultrasound at 20 to 35 kHz [20]. This discrepancy may be attributed to variations in the capacity for sample handling and power output throughout the experiment. Industrial-grade ultrasound equipment often operates at lower frequencies due to the limitations of piezoelectric ceramic sizes for high-power outputs. Elevating the ambient static pressure is recognized as an effective strategy for enhancing cavitation effects [21], [22].

Considering the research outlined above, improving the effectiveness of low-frequency ultrasound for graphite exfoliating and promoting the use of ultrasonic probes in large-scale graphene production is crucial. In this study, different ultrasonic probes were tested to examine the influence of low ultrasonic frequencies on cavitation phenomena. Furthermore, it provided an in-depth analysis of the synergistic effects of these factors and variations in static pressure on optimizing ultrasonic exfoliating efficiency.

## 2 Materials and methods

### 2.1 Sample slurry and experimental device

In this study, 45.00 g expanded graphite from Qingdao Yanhai Carbon Materials Co., Ltd. (YH-200) was used as raw material. 2.25 g polyvinylpyrrolidone (PVP) was added as a dispersant. Deionized water was added to reach a total weight of 1500.00 g, then mixed and stirred evenly to prepare the sample slurry.

**Fig. 1:**
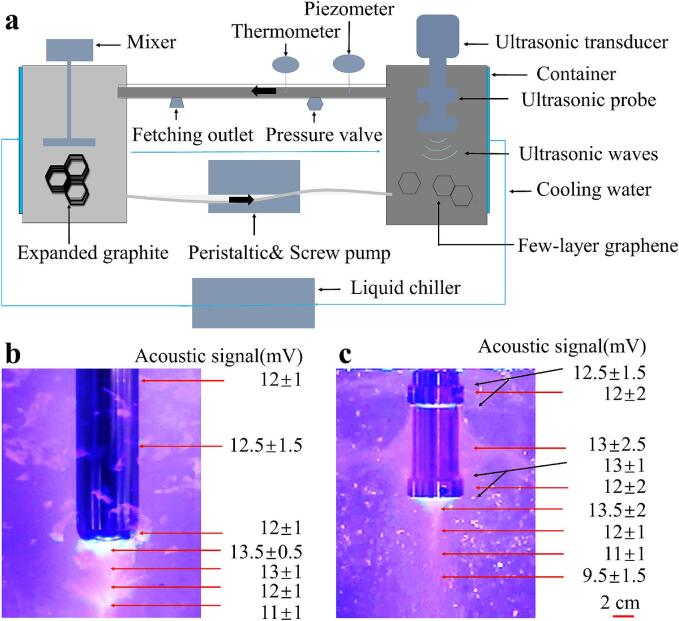
a: Schematic diagram of cyclic ultrasonic system. b and c: Ultrasonic cavitation photography of rod and dumbbell probe. The red and black arrows indicate the intensity of the acoustic signal in this area. (For interpretation of the references to colour in this figure legend, the reader is referred to the web version of this article.)

As shown in Fig. 1. a, the sample slurry was processed in a cyclic ultrasonic system to prepare FLG, with ultrasonic treatment conducted in a 2.2 L container. Throughout a 300 min experimental period, a total of 680 cycles were executed using a peristaltic pump (unpressurized) (BR −8000, Zibo Newkai Mechanical and Electrical Equipment Co., Ltd.) and a screw pump (pressurized) (YE 2–80 M2-4, ZHE JIANG XL-MOTOR Co., Ltd), maintaining a flow rate of 3.4 L/min. This dynamic procedure was maintained continuously till the experiment's conclusion. Throughout these operations, with a fixed flow rate, the static pressure within the container was modulated by adjusting the valve. To minimize experimental error, 6 ℃ cold water was utilized to control the ultrasonic environment temperature, ensuring that the sample slurry remained within 40 ℃ for optimal flow stability.

In this study, the ultrasonic system (ResoLabR-1500) manufactured by Resonance Technology (Ningbo) Co.; ltd. was selected. The vibration amplitude of the titanium alloy (Ti6Al4V) probes was adjusted to maintain the actual output ultrasonic power at 500 W. The probe's acoustic signal strength (mV) was captured in Figs. 1. b and c using an ultrasonic sound pressure sensor (HONDA, HUS-5 SUKG, Japan) [23]. Additionally, real-time voltage and current monitoring during the ultrasonic process was performed using an oscilloscope (TEKTRONIX MSO54). The actual output power was calculated using the formula P = U_rms_ × I_rms_ × cos (φ), where P is the ultrasonic power, Urms is the real-time voltage, Irms is the real-time current, and φ is the phase angle of voltage and current [13].

### 2.2 Characterization and testing of graphene

The BT-9300 laser diffraction particle size analyzer (LDPA) was employed to assess graphene's particle size and specific surface area [24]. The sample underwent a vacuum freeze-drying process before applying conductive adhesive to maintain FLG's pristine structure and reduce the risk of damage. The micromorphological features of the graphene were examined using a Hitachi S4800 field emission scanning electron microscope (SEM) with a low acceleration voltage of 4.0 kV [25]. Graphene's structure was analyzed using Renishaw Raman spectroscopy at 532 nm, positioned on a single crystal silicon substrate, with over 15 Raman spectra of FLGs randomly collected within the 1000–3000 cm^−1^ range at 50 × magnification.

To accurately determine the thickness and layer count of FLG, a diluted sample was deposited on a micro-grid copper mesh and imaged using a JEOL 2100 High-Resolution Transmission Electron Microscope (TEM) at 200 kV. Each sample was documented with over 20 high-resolution images to minimize measurement errors. FLG dimensions and layer count were quantified using ImageJ and Digital Micrograph software. A 10 × 10 mm silicon oxide wafer with a 300 nm oxide layer was used as the sample substrate. Additionally, CSPM5500 Atomic Force Microscopy (AFM) characterization was performed with the AFM in tapping mode at a scanning rate of 1.5 Hz and 256 samples per line. The substrate was cleaned with ethanol in a 250 W ultrasonic bath for 3 min. The sample was dispersed for 5 min, then diluted with ethanol to achieve an ultra-light gray transparency. It was drop-cast onto the substrate and dried in a vacuum oven at 35 ℃ for 20 min. The NanoScope software was used to process AFM images, and each sample counts more than 45 measurement points to estimate the average thickness to ensure the credibility of the results.

To evaluate the electrical properties of FLG, FLG-CMC composite films were fabricated on Polyethylene Terephthalate (PET) substrates using a 1:9 mass ratio of carboxymethyl cellulose (CMC) to FLG via a coating process. According to the four-probe method, the thickness of the thin film was measured using a thickness gauge, while square resistance measurements determined its resistance. Based on Ohm's law, the van-der-Pauw method was calculated to the composite conductivity of FLG according to Formula (1) [26], with an approximate error of 8 %. Where σ is the conductivity, S/m; r is the membrane resistance, Ω; b is the width between the measuring points, 1 mm; l is the distance between the voltage segments of the measurement point, 1 mm; d is the thickness of the measuring point, mm.

(1) $$\sigma =R^{-1}∙b∙d∙l$$

## 3 Result and discussion

### 3.1 Ultrasonic probe types

Figs. 1. b and c display the cavitation cloud of the rod and dumbbell probes in water and the intensity of the acoustic signal (the voltage value measured by the hydrophone). Both probes exhibit a strong correlation between the density of the bubble clouds and the acoustic signal intensity. In Fig. 1. b, numerous irregular bubble clouds are observed around the cylindrical section of the rod probe, with a relatively uniform sound intensity distribution. In contrast, a dense cylindrical bubble cloud forms beneath the tip, where the sound intensity reaches its maximum. The unique structure of the dumbbell probe, as depicted in Fig. 1. c, not only produces regular sound waves from its cylindrical sections similar to the rod probe but also generates a pronounced ultrasonic interference zone in the middle section. This interference arises from the interaction between reverse vertical and horizontal sound waves. Consequently, both ends, and the middle of the dumbbell probe exhibit strong acoustic signals, leading to an overall “干” shape of the bubble cloud, with a weaker upper region. Additionally, due to structural influences, a conical bubble cloud is observed at the lower end of the probe.

**Fig. 2:**
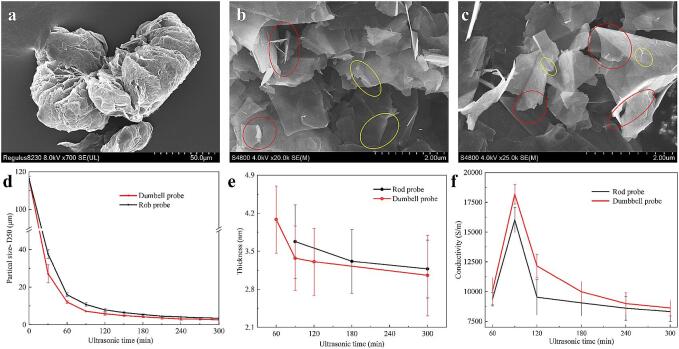
SEM images: a, expanded graphite raw material; b and c, after 300 min of ultrasound, the FLG was prepared by the rod and dumbbell probe. Red marks indicate rippled, crumpled, and curled areas; yellow marks highlight regions with crease lines and wrinkles. Evolution of FLG characteristics with increasing ultrasonic time: d, particle size(D50); e, thickness; f, the conductivity of FLG-CMC films. (For interpretation of the references to colour in this figure legend, the reader is referred to the web version of this article.)

The morphology, size, thickness, and conductivity of the graphene prepared by the rod and dumbbell probes are detailed in Fig. 2. SEM images showed that the expanded graphite with an initial particle size of > 100 μm was broken and exfoliated into FLG of about 3 μm after ultrasonic treatment for 300 min. The FLGs produced by both probes displayed distinct curling and folding characteristics [25], [27]. However, the FLG produced with the dumbbell probe displayed more pronounced three-dimensional wrinkling, indicating that it possesses thinner and more flexible properties. This observation is supported by the AFM characterization results in Fig. 2. e. Analysis of the data in Figs. 2. d-f reveals that, with increased ultrasonic treatment time, the particle size and thickness of the FLG decrease progressively. Concurrently, the conductivity of FLG-CMC films exhibits an initial rise followed by a decline. The dumbbell probe exhibited higher breaking and exfoliating efficiency than the rod probe during the cavitation exfoliating of graphite flakes at the same ultrasonic duration. This capability enables the dumbbell probe to produce low-thickness FLG with high conductivity efficiently, advancing its potential applications in electrical and sensing technologies [27].

Under identical frequency and power conditions, the resonance size of the cavitation bubbles generated by both probes should be consistent [28]. This indicates that the spatial distribution of bubbles becomes the main factor affecting the cavitation effect. As shown in Fig. 1, a strong acoustic signal facilitates the generation of a dense bubble cloud, and the expansion of this bubble cloud's range increases the cavitation density within the container, thereby inducing a more intense cavitation effect. This enhancement amplifies the ultrasound's effectiveness in breaking and exfoliating graphite flakes.

Consequently, the dumbbell probe, with its stronger acoustic signal and a wider range of dense bubble clouds, demonstrates superior efficiency in the preparation of FLG compared to the rod probe. We employed this efficient dumbbell probe to systematically investigate the effects of low-frequency ultrasound on FLG exfoliation.

### 3.2 Ultrasonic frequencies

**Fig. 3:**
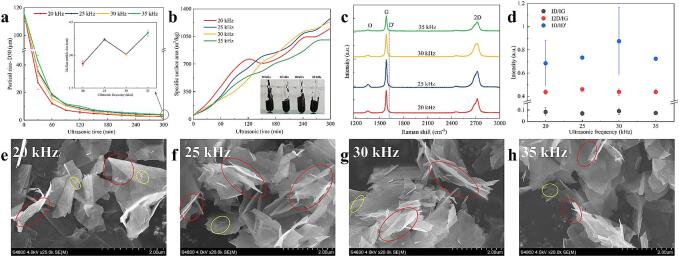
Change of FLG with ultrasonic time: a, particle size; b, specific surface area (the lower right corner is the FLG dispersion). FLG of ultrasonic 300 min: c, Raman spectrum; d, the ratio of Raman characteristic peaks. e-h, SEM images of FLG prepared at different frequencies. Red marks indicate rippled, crumpled, and curled areas; yellow marks highlight regions with crease lines and wrinkles. (For interpretation of the references to colour in this figure legend, the reader is referred to the web version of this article.)

As illustrated in Fig. 3. a and b, both the particle size and thickness of the graphite flakes continuously decreased with increasing treatment time during ultrasonication, approaching the exfoliation limit at 300 min. Notably, while there is no positive correlation between the particle size of FLGs and the ultrasonication frequency, the larger FLG produced at 25 kHz has the highest specific surface area.

The SEM images in Fig. 3. e-h further confirm the advantages of the 25 kHz preparation in terms of thickness, clearly displaying the size of the FLGs alongside the distribution of wrinkled and curled areas. Research indicates that prolonged ultrasonic treatment degrades the quality of graphene sheets and increases structural defects [12]. Therefore, a detailed characterization of the FLGs prepared via 300 min of ultrasonic treatment is crucial for assessing their structural integrity.

Fig. 3. c presents the Raman spectra of different FLGs, which exhibit the characteristic graphene peaks: the D peak at approximately 1350 cm^−^^1^, the G peak near 1580 cm^−^^1^, the D' peak around 1620 cm^−^^1^, and the 2D peak near 2700 cm^−^^1^ [29]. The weak D and almost invisible D peaks indicate that the four FLGs belong to low-defect graphene with serrated edges, showcasing high crystallinity [30].

Fig. 3. d presents the data distribution of the characteristic peak intensity ratios, showing that the ID/IG ratio is below 0.1 and the ID/ID' ratio is below 3.5, which confirms that the FLGs exhibit only minor edge defects [31]. Among these, the FLG was subjected to 25 kHz ultrasonic treatment, which exhibits the lowest ID/IG ratio and demonstrates superior quality. This suggests that ultrasound primarily disrupts the lattice structure between carbon atoms during the FLG preparation and does not excessively deform carbon bonds or cause excessive sp^3^ hybridization. The I2D/IG ratio is also closely associated with the number of graphene layers [18]. Backes et al. [32] have demonstrated that Formula (2) can theoretically determine the number of layers in FLG. In the formula M_1_ = I2D/IG, the error is ∼ 25 %. The theoretical average layers of the four FLGs prepared at 20–35 kHz are 7.1 ± 0.7, 6.3 ± 0.8, 7.1 ± 1.3 and 7.1 ± 1.1, respectively, which are similar to the specific surface area distribution.

(2) $$N=1.04∙{M_{1}}^{-2.32}$$

Although it is preliminarily confirmed that 25 kHz can prepare the thinnest FLG with the smallest damage, its specific exfoliating advantage still needs further quantitative verification.

**Fig. 4:**
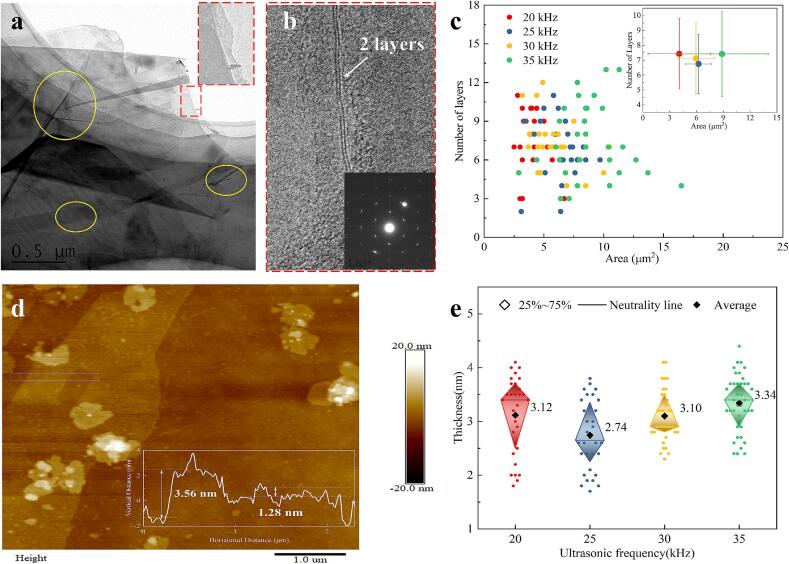
FLG prepared by ultrasonication for 300 min. a: TEM image of FLG prepared at 25 kHz (red dotted line is the local amplification, yellow circle is the crease). b: Local high-resolution TEM images (the lower right corner is the diffraction pattern of the region). c: TEM characterization results. d: AFM image of FLG prepared at 25 kHz. e: AFM characterization results. (For interpretation of the references to colour in this figure legend, the reader is referred to the web version of this article.)

TEM is excellent in directly observing the morphology and number of layers of FLG. The scatter plot in Fig. 4. c reveals that the areas of the four FLGs predominantly fall within the range of 3–10 μm^2^, with the number of layers generally ranging from 4 to 10. The average area of these FLGs is consistent with the size distribution results obtained by LDPA and SEM. In terms of the average number of layers, although the difference between the four FLGs is within the margin of error, the following trend is still observed: FLG_25 kHz_＜FLG_30 kHz_＜FLG_35 kHz_＜FLG_20 kHz_.This result not only verifies the accuracy of the theoretical layer number of Raman calculations but also aligns with previous studies [20], confirming the superior exfoliation effect of the 25 kHz frequency.

However, the TEM images in Fig. 4. a and b, reveal potential inaccuracies from localized edge coverage and surface irregularities, such as wrinkles, in the enlarged FLG specimens. This highlights the necessity of employing AFM for a more detailed assessment of FLG thickness. Comparing AFM results with TEM data will provide a more comprehensive evaluation of the graphene layer count and enable more accurate quantification of the exfoliation effect achieved through ultrasound.

Fig. 4. d presents AFM characterization images of the FLG samples. The scatter distribution in Fig. 4. e reveals that the thickness of the FLGs is predominantly clustered around 3 nm, with a maximum deviation not exceeding 4.5 nm. Notably, the FLG prepared at 25 kHz exhibited both the lowest average and median thickness. Reference [33] stipulates that the thickness of monolayer graphene on a silicon oxide substrate range between 0.8 to 1.2 nm, while the interlayer spacing within graphite is approximately 0.34 nm. Therefore, the number of graphene layers can be calculated using Formula (3), where n is the number of layers, h is the height (nm) characterized by AFM, and h_1_ is the thickness of monolayer graphene (0.8–1.2 nm). Through analysis predicated on the varying ultrasonic frequencies employed during synthesis, the average number of layers of the four FLGs was calculated to be 7.3 ± 0.6, 6.1 ± 0.6, 7.2 ± 0.6 and 7.9 ± 0.3, respectively, and the error with the TEM characterization results was within 7 %.

(3) $$n=\left[\left(h-h_{1}\right)/0.34\right]+1$$

Overall, within the commonly used frequency range of 20–35 kHz, the 25 kHz frequency demonstrated the capability to produce FLGs with the fewest layers, minimal defects, and larger sizes when ultrasonically breaking down graphite flakes to their limits and minimizing the variance of the FLGs. This indicates that, under identical conditions, 25 kHz represents the optimal choice for FLG preparation within this frequency range.

In ultrasonic exfoliation, cavitation bubbles are generated and collapsed when the ultrasonic sound pressure surpasses a critical threshold. This process induces mechanical, sonochemical, and thermal effects on the graphite sheet, leading to its fragmentation and exfoliating, ultimately producing graphene [34], [35]. Thus, the size, number, and distribution of cavitation bubbles are crucial factors that influence cavitation intensity [36].

When the resonance frequency of a cavitation bubble aligns with the ultrasonic frequency, the cavitation effect is maximized, and the bubble achieves its resonance radius. By applying the Minnaert equation [18], [37], [38], the resonance radius (R) of cavitation bubbles can be determined for various ultrasonic frequencies, assuming constant liquid density (ρ), the ratio of isobaric to isochoric heat capacity of the gas (γ), and the ambient pressure surrounding the bubble (P_A_). This relationship is detailed in Formula (4). Considering the radius of ∼ 20 kHz cavitation bubbles in water is 138.2 μm [39], the radius of cavitation bubbles at four frequencies ranging from 20-35 kHz can be calculated as 138.2 μm, 111.6 μm, 92.1 μm, and 79.0 μm, correspondingly.

(4) $$R={\left(2\pi f\right)}^{-1}{∙\left(3\gamma P_{A}\right)}^{0.5}∙\rho^{-0.5}$$

Analysis of the literature indicates that, for constant ultrasonic power, an increase in ultrasonic frequency leads to a decrease in both the concentration and radius of cavitation bubbles [14], [19]. Within the 20–50 kHz frequency range, transient cavitation, driven by the collapse of these bubbles, becomes the dominant effect [40]. The energy released during cavitation is proportional to the bubble radius [41]. Hence, FLG with the smallest and largest particle sizes was produced using 20 kHz for the largest cavitation bubble radius and density and 35 kHz for the smallest cavitation bubble radius and density, respectively. However, the sizes and thicknesses of FLG prepared at 25 kHz and 30 kHz did not fully conform to these trends.

**Fig. 5:**
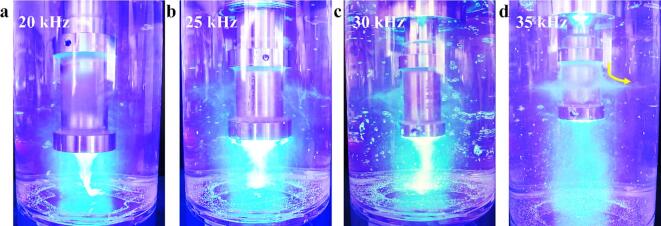
a-d: Cavitation photographies at different ultrasonic frequencies (device model: xiaomi 14-lieica).

As shown in Fig. 5, higher ultrasonic frequencies lead to a more densely distributed bubble cloud near the middle of the probe. Notably, the 25 kHz probe generates a larger and denser bubble cloud at its lower end compared to the 30 kHz and 35 kHz probes. Therefore, 25 kHz is the optimal frequency for producing FLG with larger particle sizes, fewer layers, and reduced defects within the 20–35 kHz frequency range.

### 3.3 The static pressure of ultrasonic environment

According to Formula (4), applying static pressure can enhance the internal pressure of cavitation bubbles, in addition to adjusting ultrasonic parameters [42], [43]. This procedure expands the bubble's resonance radius [44] and elevates the cavitation threshold [45], thereby amplifying the energy released during bubble collapse and strengthening the cavitation erosion effect [13]. Consequently, a static pressure of 0.2 MPa was applied to prepare FLG under the 25 kHz dumbbell probe condition, and the impact of increasing the cavitation bubble radius (46.1–58.6 μm) on the exfoliating process was investigated.

**Fig. 6:**
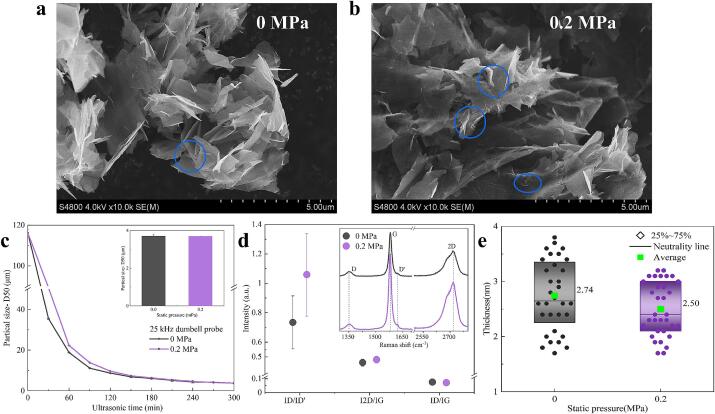
FLG prepared by 25 kHz dumbbell probe ultrasound with different static pressures for 300 min. a and b: SEM images of different FLGs. Blue marks the ripples of FLG. c, the change of FLG particle size with ultrasonic time. d: Raman spectrum and characteristic peak ratio of FLG. e: AFM characterizes the thickness box of FLG. (For interpretation of the references to colour in this figure legend, the reader is referred to the web version of this article.)

The SEM images in Fig. 6 illustrate that FLG prepared under a static pressure of 0.2 MPa exhibits more pronounced ripple characteristics than other FLG, despite having similar three-dimensional structures. This indicates that the FLG synthesized at 0.2 MPa has a softer texture and fewer layers. Data in Fig. 6. c reveals that, although the limit particle sizes of FLG are comparable, increased static pressure markedly reduces the breaking efficiency of the cavitation effect on graphite sheets during the initial 150 min of ultrasonic treatment.

In Fig. 6. d, the FLG prepared under pressurization shows a pronounced and sharper 2D peak, initially suggesting fewer layers. Further analysis of the characteristic peak ratios reveals that an ID/ID' value approaching 1 signifies that both FLG samples, prepared with and without pressurization, exhibit primarily edge defects with minimal deformation in the carbon bond structure [31]. Notably, the FLG prepared at 0.2 MPa exhibits a reduced ID/IG ratio, indicative of a lower defect density. According to Formula (2), the estimated average number of layers for FLG prepared at 0 MPa and 0.2 MPa are 6.3 and 5.7, respectively, with an error margin of approximately 25 %.

Further characterization of the FLG was performed using AFM, with the average layer number calculated according to Formula (3). The results showed that the actual average layer numbers of FLG produced under 0 MPa and 0.2 MPa were 6.1 ± 0.6 and 5.4 ± 0.6, respectively, closely aligning with the expected values from Formula (2). Fig. 6. e indicates that applying pressure significantly reduced the overall thickness of the FLG and resulted in a more concentrated thickness distribution. This finding suggests that although the enhancement of ultrasonic cavitation effect is not obvious at a pressure of 0.2 MPa, the increase of cavitation bubble size and the increase of cavitation threshold are helpful to improve the ultrasonic stripping effect. Consequently, this improvement leads to greater thickness uniformity of FLG and a reduction in defect density.

**Fig. 7:**
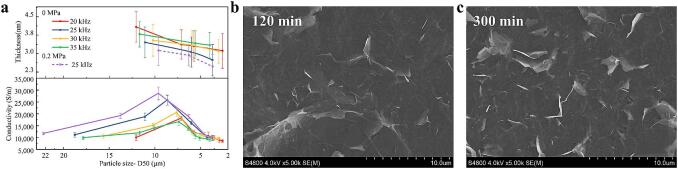
a: Afm characterization results and conductivity changes of flg prepared at different frequencies. b and c: FLG-CMC films prepared by 25 kHz dumbbell probe ultrasonic 120 min and 300 min at 0 MPa.

Graphene's exceptional electrical properties render it valuable for applications in solar energy [46], capacitors [47], and electromagnetic shielding [48]. Despite the impact of defects [49], size [50], [51], and thickness [52] influence the electrical properties of graphene, Raman spectroscopy of FLG indicates that edge defect density does not affect conductivity. As illustrated in Fig. 7. a, the conductivity of the FLG-CMC film initially increases and then decreases with reductions in both FLG particle size and thickness. Remarkably, under normal pressure ultrasonic treatment, FLG prepared at 25 kHz exhibited the highest conductivity within the 20–35 kHz range. After pressurized ultrasonic treatment, the conductivity of FLG prepared at 25 kHz reached 2.86 × 10^4^ S/m, which surpasses that of most graphene-based composites [53]. It is essential to note that when the FLG particle size is constant, lower FLG thickness results in higher conductivity; similarly, at the same FLG thickness, larger particle size corresponds to increased conductivity.

The conductivity of graphene is intricately linked to its structural properties. The sp^2^ hybridization between carbon atoms introduces additional π electrons delocalized at room temperature, resulting in high conductivity [54]. While CMC can enhance the adhesion of FLG, it can also impede electron movement and disrupt current transfer [55]. In FLG-CMC films of identical area, those with larger particle sizes and reduced thickness exhibit enhanced conductivity. This improvement is attributed to the reduced stacking and overlapping of FLG, which leads to decreased CMC content at the junctions. Consequently, this reduction in CMC content decreases electrical interference and improves surface smoothness. As demonstrated in Figs. 7. b and c, the films with higher conductivity exhibit smoother surfaces and fewer protrusions.

## 4 Conclusion

In this study, we systematically examined the effects of probe type and ultrasonic frequency on the cavitation effect, optimizing the ultrasonic exfoliating process of flake graphite through the introduction of static pressure. Throughout the experimental and characterization processes, the samples were not subjected to any screening. Furthermore, the analysis of various FLGs produced via the ultrasonic method yielded several significant findings:

1. Comparative analysis of rod and dumbbell probes revealed that the dumbbell probe can enhance both the cavitation breaking and exfoliation efficiencies of graphite flakes due to its larger strong acoustic field region.
2. Within the frequency range of 20–35 kHz, the 25 kHz frequency facilitates the preparation of the thinnest large-size FLGs while minimizing damage to the graphene structure, thereby making it the optimal choice for exfoliating graphite flakes.
3. Experiments utilizing the 25 kHz dumbbell probe at a pressure of 0.2 MPa resulted in a reduction in thickness distribution and defect density of the FLGs, alongside a notable increase in conductivity.

## CRediT authorship contribution statement

**Minhui Gao:** Writing – original draft, Software, Methodology, Investigation, Formal analysis, Conceptualization. **Hu Zong:** Investigation, Data curation, Conceptualization. **Lei Yu:** Validation, Resources, Methodology. **Jiacheng Yao:** Investigation. **Su Zhao:** Writing – review & editing, Supervision, Project administration, Funding acquisition, Conceptualization. **Ying Zhou:** Supervision, Project administration, Funding acquisition, Conceptualization. **Yifei Li:** Resources, Methodology. **Yanyuan Zhou:** Resources, Methodology. **Jiahao Zhang:** Resources, Methodology. **Ronghe Li:** Investigation.

## Declaration of competing interest

The authors declare that they have no known competing financial interests or personal relationships that could have appeared to influence the work reported in this paper.
